# The determination of peanut (*Arachis hypogaea* L.) pod-sizes during the rapid-growth stage by phytohormones

**DOI:** 10.1186/s12870-023-04382-w

**Published:** 2023-07-26

**Authors:** Zhenghao Lv, Dongying Zhou, Xiaolong Shi, Jingyao Ren, He Zhang, Chao Zhong, Shuli Kang, Xinhua Zhao, Haiqiu Yu, Chuantang Wang

**Affiliations:** 1 College of Agronomy, Peanut Research Institute, Shenyang Agricultural University, Shenyang, China; 2 Shandong Peanut Research Institute, Qingdao, China

**Keywords:** Peanut (*Arachis hypogaea* L.), Pod size, Pod growth, Cytological analysis, Transcriptome, Phytohormone

## Abstract

**Background:**

Pod size is an important yield target trait for peanut breeding. However, the molecular mechanism underlying the determination of peanut pod size still remains unclear.

**Results:**

In this study, two peanut varieties with contrasting pod sizes were used for comparison of differences on the transcriptomic and endogenous hormonal levels. Developing peanut pods were sampled at 10, 15, 20, 25 and 30 days after pegging (DAP). Our results showed that the process of peanut pod-expansion could be divided into three stages: the gradual-growth stage, the rapid-growth stage and the slow-growth stage. Cytological analysis confirmed that the faster increase of cell-number during the rapid-growth stage was the main reason for the formation of larger pod size in Lps. Transcriptomic analyses showed that the expression of key genes related to the auxin, the cytokinin (CK) and the gibberellin (GA) were mostly up-regulated during the rapid-growth stage. Meanwhile, the cell division-related differentially expressed genes (DEGs) were mostly up-regulated at 10DAP which was consistent with the cytological-observation. Additionally, the absolute quantification of phytohormones were carried out by liquid-chromatography coupled with the tandem-mass-spectrometry (LC–MS/MS), and results supported the findings from comparative transcriptomic studies.

**Conclusions:**

It was speculated that the differential expression levels of *TAA1* and *ARF* (auxin-related), *IPT* and *B-ARR* (CK-related), *KAO*, *GA20ox* and *GA3ox* (GA-related), and certain cell division-related genes (*gene-LOC112747313* and *gene-LOC112754661*) were important participating factors of the determination-mechanism of peanut pod sizes. These results were informative for the elucidation of the underlying regulatory network in peanut pod-growth and would facilitate further identification of valuable target genes.

**Supplementary Information:**

The online version contains supplementary material available at 10.1186/s12870-023-04382-w.

## Background

Cultivated peanut (*Arachis hypogaea* L.) can provide humans with nutrients such as protein and essential fatty acids [1], and is widely cultivated worldwide as an important oilseed crop and cash crop. In recent years, peanut has become one of the three major oilseed crops in China, which plays an important role in ensuring the safety of edible oil in China [2]. Yield potential has always been a vital target of plant breeding in peanut, and the pod size directly influences the final yield and quality of peanut. The peanut pod is composed of shell and seed. Swelling of the shell can affect potential yield [3]. Larger shells provide more room for development and are more likely to obtain larger seeds [4]. Nevertheless, the sizes of seeds and organs are not always positively correlated because they have separate regulatory pathways [5]. For peanut, previous studies have shown that pod-growth can be divided into two stages of pod expansion and seed filling [6]. Pod expansion is mainly performed at the early-growth stage (10DAP-30DAP), during which the pod reaches its final size. Therefore, studies on the peanut pod-growth during this critical period might be helpful to understand the determination-mechanism of peanut pod sizes.

Plant fruit development is mainly controlled by cell division and cell expansion [7]. And fruit size is determined by the number and size of cells [8]. Rice *OsSPL16* encodes a protein that is a positive regulator of cell proliferation. The high expression of this gene promotes cell division and generates wider grain [9]. On the contrary, restriction of cell proliferation produces smaller organs and seeds [5, 10]. Moreover, studies suggest that the grain size of rice could be modulated by increasing cell expansion in spikelet hulls [11, 12]. However, the regulation of cell-number and cell-size are often controlled and coordinated by the mechanism of regulating plant and organ size. Changing either determinants does not necessarily change the final organ size [13]. At present, it is still unclear whether the difference in pod size of peanut is caused by the difference in cell number or cell size.

Phytohormones play essential roles in the regulation of pod-growth, pod size and crop yield. Auxin is a critical phytohormone that plays crucial roles in embryogenesis, organogenesis, cell determination and division, flower and fruit development [14]. The auxin biosynthesis mutation of garden pea resulted in small seeds, and the phenotypic effect of the mutation was partially reversed by auxin application [15]. The growth and development of peanut pegs and pods are regulated by auxin [16]. The pod weight and yield per pod treated by indole-3-acetic acid (IAA) and auxin polar transportinhibitor 2,3,5-triiodobenzoic acid (TIBA) were significantly increased, indicating that auxin may increase yield by promoting pod development [17]. Cytokinin (CK) regulates plant growth and development and plays a key role in regulating cell proliferation [18]. The *AtENO2* mutant reduces seed size by decreasing the content of cytokinin [19]. Furthermore, the triple cytokinin receptor mutant produces larger seeds [20]. Studies have shown that cytokinin and auxin can synergistically promote cell division and thus influence fruit size [21, 22]. Gibberellin (GA) is a phytohormone that promotes cell division and elongation and participate in many developmental processes [23]. DELLA proteins (aspartic acid–glutamic acid–leucine–leucine–alanine) inhibit plant growth by reducing both cell proliferation and expansion rates [24]. GA promotes cell division and expansion by inhibiting the activity of DELLA proteins [25, 26]. In rice, *miR396ef* mutation promotes the increase in the level of GA precursor, thereby promoting the biosynthesis of GA, and improving grain yield by increasing grain size [27]. In peanut, *AhGRF5a* and *AhGRF5b* are response factors to GA3 and express higher levels in pod. These two genes may play key roles in peanut pod-growth [6].

Currently, several studies on transcriptome research related to peanut pod-growth have been reported [3, 28–31]. However, few studies have focused on phytohormones regulation pod-growth during early-stage. In the present study, cytological observation, RNA-Seq and LC–MS/MS for absolute phytohormones quantification were performed to explore the physiological and molecular changes of peanut pod during this period. The results will facilitate understanding the decisive role of a series of changes during early-growth stage on the formation of peanut pod size, and be helpful for cloning of candidate genes and molecular breeding.

## Results

### Pod differences between Tif and Lps during early-growth stage

**Fig. 1:**
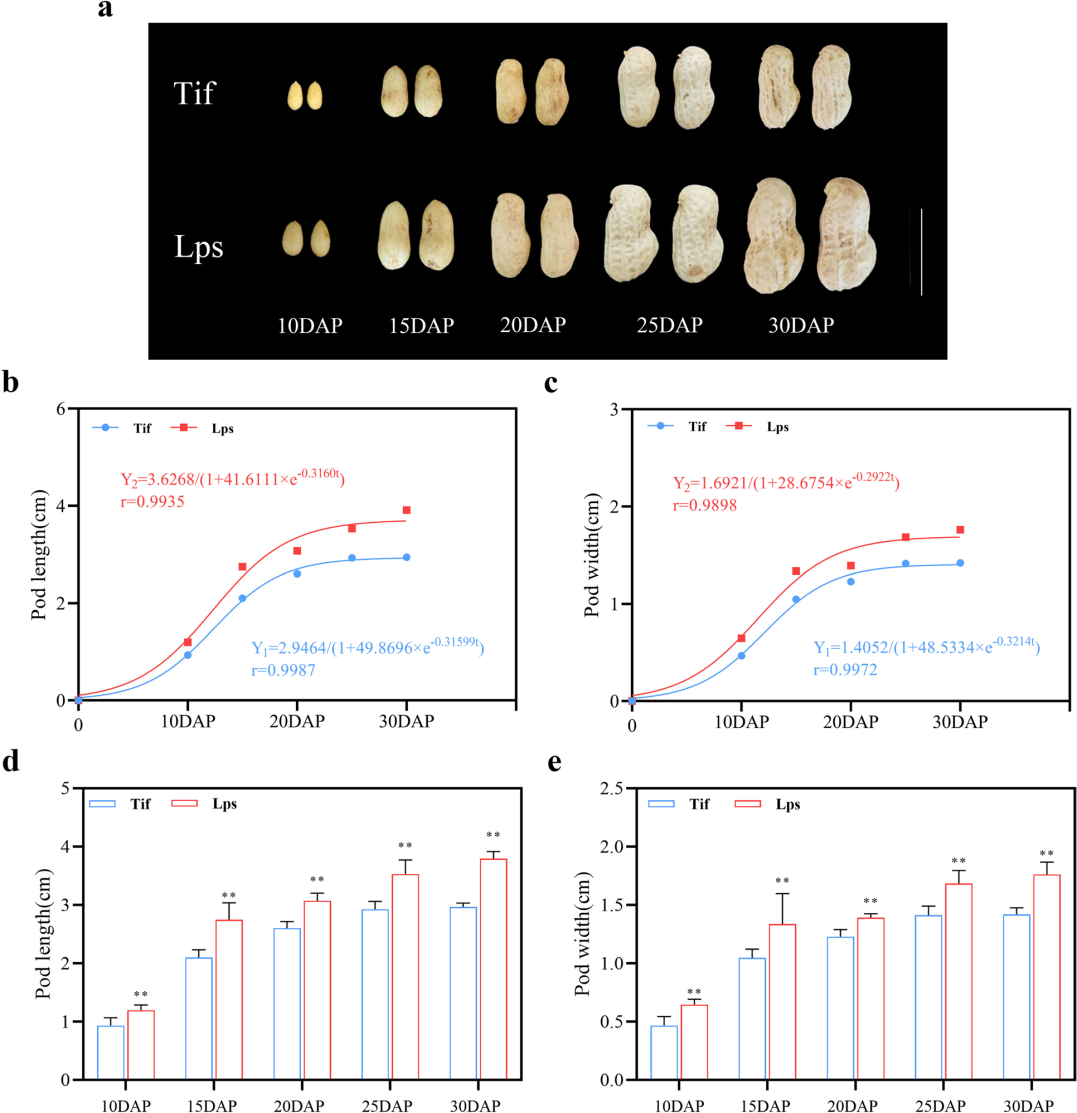
Phenotypes of Tif and Lps during the early-growth stage. **a** The phenotypic characteristics of Tif and Lps pods at five different growth stages. **b** Observed and fitted the pod length of Tif and Lps using logistic growth function. **c** Observed and fitted the pod width of Tif and Lps using logistic growth function. **d** Pod length of Tif and Lps during early-growth stage. **e** Pod width of Tif and Lps during early-growth stage. Scale bar = 2 cm in (a). Error bar is SD. ** *p* < 0.01

In general, peanut pods begin to expand at 10DAP and reach the final size during 20DAP to 30DAP. In this study, the pod length and width of Tif and Lps were measured at 10, 15, 20, 25, and 30 days after pegging (DAP), respectively. The results showed that pods of both Tif and Lps developed rapidly from 10 to 15DAP. Subsequently, pod reticulation appeared at 25DAP and reached the final size in about 25DAP to 30DAP (Fig. 1a). Notably, the pod length and width of Lps still increased significantly during DAP25-DAP30 compared to Tif. The growth curve of the peanut pods was close to the S type, and the pod length and width could be well fitted by a logistic growth curve (Fig. 1b, c). Calculations showed that the pod length and width of both Tif and Lps increased fastest during about 8DAP to 16DAP (*t*_*1*_-*t*_*2*_). The time of maximum rates (*Tm*) of both pod length and width of Tif was about 12DAP, and the *Tm* of pod length and width of Lps was about 12DAP and 11DAP, respectively (Table S2). In addition, the pod length and width of Lps were significantly greater (*p* < 0.01) than those of Tif at each growth stage (Fig. 1d, e).

### Distinct cell number and cell size between Tif and Lps

**Fig. 2:**
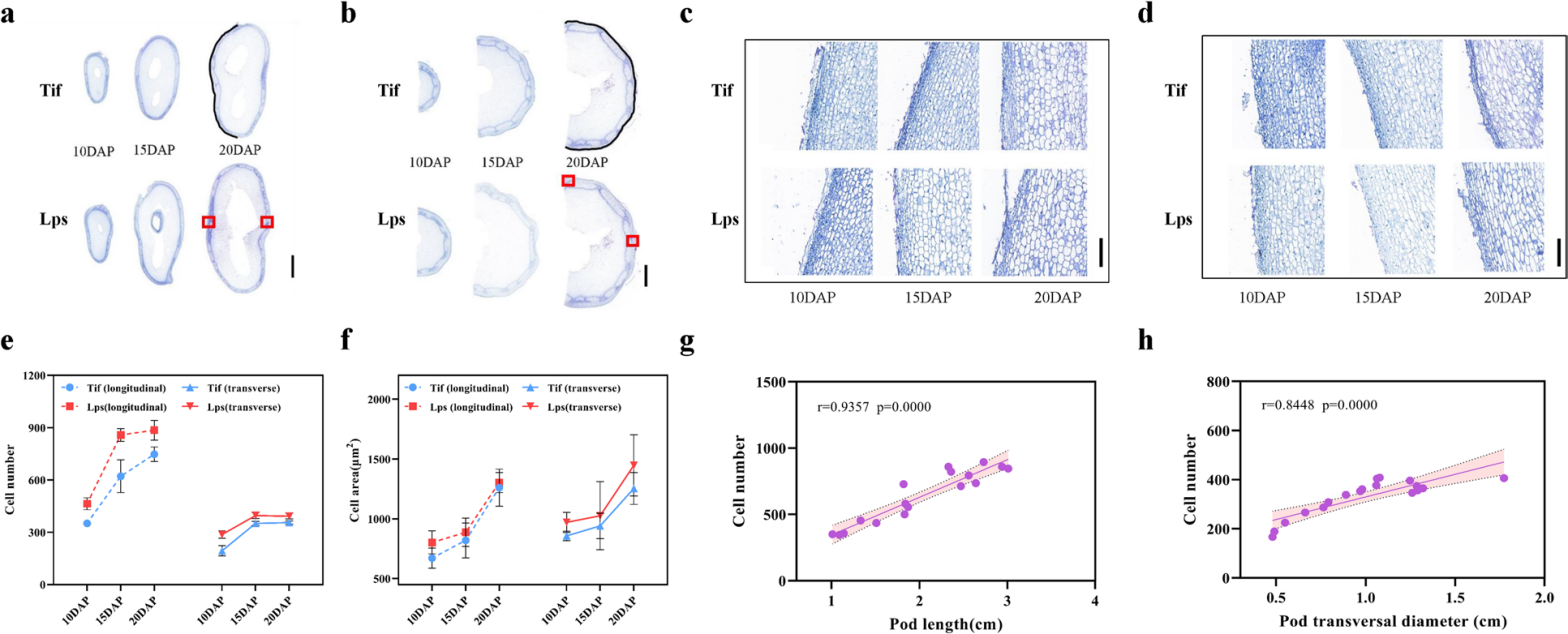
Differences in cell development between Tif and Lps lines. **a** The longitudinal sections of shell of Tif and Lps at three growth stages. **b** The transverse sections of shell of Tif and Lps at three growth stages. **c** The longitudinal sections of Tif and Lps at three growth stages. **d** The transverse sections of Tif and Lps at three growth stages. **e** Cell number of longitudinal sections and transverse sections in Tif and Lps at three growth stages. **f** Cell area of longitudinal sections and transverse sections in Tif and Lps at three growth stages. **g** Correlation between pod length and cell number. **h** Correlation between pod transversal diameter and cell number. Error bar is SD. Scale bar = 5000 μm in (**a**); scale bar = 2000 μm in (**b**); scale bars = 200 μm in (**c**) and (**d**)

Since the pod size of Tif and Lps changed significantly from 10 to 20DAP, we performed histological analysis in longitudinal and transverse sections of the shell at 10DAP, 15DAP, and 20DAP, respectively. Cell number was calculated along the black lines in one single-cell line. Cells calculated in this way were regarded as cell numbers in longitudinal or transverse sections. Cell areas in the red box were calculated as the cell areas of the longitudinal or transverse section (Fig. 2a, b). The cell number of Tif and Lps increased rapidly during 10DAP to 15DAP. In the longitudinal section of each stage, the cell-number of Lps was 32.0%, 38.1% and 18.4% more than that of Tif, respectively (Fig. 2e). For the transverse section, the cell-number in Lps was significantly more (*p* < 0.05) than that of Tif at 10DAP, 15DAP and 20DAP by 48.19%, 11.69% and 10.32%, respectively (Fig. 2e). The cell-area of Tif and Lps increased rapidly during 15DAP to 20DAP. The cell-area of Lps was significantly greater (*p* < 0.05) than that of Tif only on the longitudinal of 10DAP and transverse sections of 20DAP (Fig. 2f).

Based on the investigation, we found that both cell number and cell size of Lps were greater than that of Tif during the early-growth stage. Herein, we analyzed the relationship between pod length and pod transversal diameter with cell number and cell area, respectively. In both longitudinal and transverse sections, pod length and transversal diameter were significantly correlated with cell number and cell area (*p* < 0.01), indicating that the growth of pod was promoted by the increase of cell number and area. For the longitudinal section, pod length showed a strong linear relationship (r = 0.9357) with cell number (Fig. 2g). However, compared with the number of cells, the linear relationship between pod length and cell area is weak(r = 0.6725) (Fig. S1a). For the transverse section, a strong linear relationship (r = 0.8448) was found in the analysis of pod transversal diameter vs. cell number (Fig. 2h), not in pod transversal diameter vs. cell area (r = 0.6834) (Fig. S1b). Therefore, the pod length and width were mainly determined by the cell number.

### Transcriptome sequencing

**Fig. 3:**
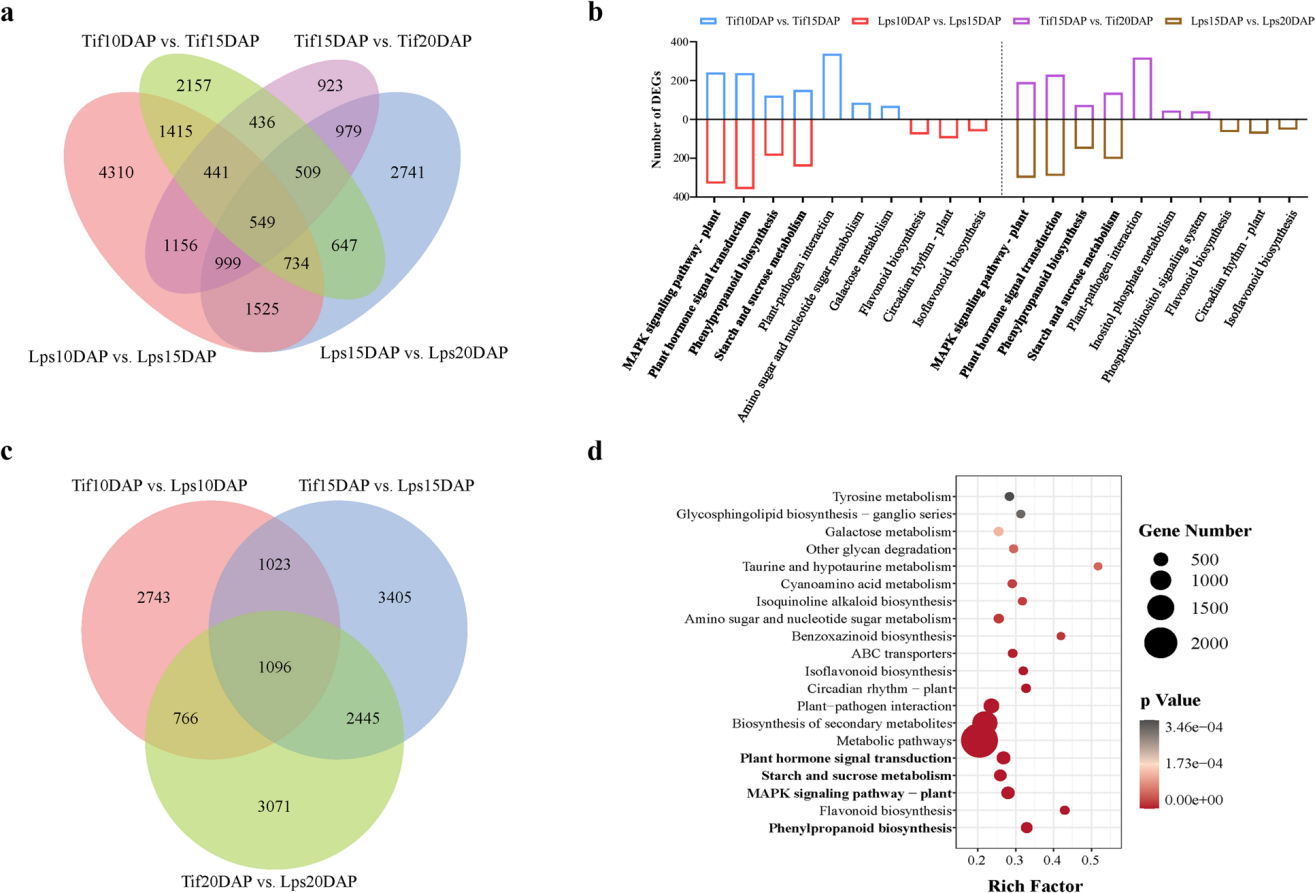
DEGs of Tif and Lps at 10DAP, 15DAP and 20DAP. **a** Venn diagram of genes in Tif and Lps in four comparison groups. **b** KEGG enrichment analysis of Tif and Lps in four comparison groups. **c** Venn diagram of genes in Tif and Lps at three growth stages. **d** KEGG enrichment analysis of Tif and Lps at three growth stages

The transcriptome sequencing was performed for peanut shell at 10DAP, 15DAP and 20DAP. After removing the low-quality reads, a total of 853,569,042 clean reads were obtained. The percentages of Q30 and GC were 93.38–94.28% and 44.64–45.23%, respectively, indicating that the quality of transcriptome sequencing data is high. Gene expression of Tif and Lps pods during early-growth stage was compared. There were 6888 (Tif10DAP vs. Tif15DAP), 5992 (Tif15DAP vs. Tif20DAP), 11,129 (Lps10DAP vs. Lps15DAP) and 8683 (Lps15DAP vs. Lps20DAP) genes identified as DEGs (Fig. 3a). The results showed that during 10DAP to 15DAP, there were more DEGs in Tif (Tif10DAP vs. Tif15DAP) and Lps (Lps10DAP vs. Lps15DAP), and after that, the number of DEGs decreased. During 10DAP-15DAP, GO enrichment analysis (Table S3) revealed that DEGs of Tif were mainly enriched in cell wall polysaccharide metabolic process (GO: 0010383), cell wall macromolecule metabolic process (GO: 0044036) and hemicellulose metabolic process (GO: 0010410). For Lps, DNA packaging complex (GO: 0044815), protein-DNA complex (GO: 0032993) and nucleosome (GO: 0000786) were the main enriched terms. During 15DAP-20DAP, the term response to chitin (GO: 0010200) was the most enriched in Tif, followed by microtubule binding (GO: 0008017) and tubulin binding (GO: 0015631). Meanwhile, DEGs of Lps were mainly enriched in response to chitin (GO: 0010200), trihydroxystilbene synthase activity (GO: 0050350) and plant-type cell wall (GO: 0009505). The enrichment analysis of KEGG pathways showed that the DEGs of both Tif and Lps were significantly enriched in several major metabolic pathways at each comparison group, including plant hormone signal transduction, mitogen-activated protein kinase (MAPK) signaling pathway-plant, starch and sucrose metabolism and phenylpropanoid biosynthesis (Fig. 3b; Table S4).

Further analysis showed that there were 5628, 7969 and 7378 genes identified as DEGs between Tif and Lps at 10DAP, 15DAP and 20DAP, respectively (Fig. 3c). Among them, 1096 genes were overlapped DEGs. The annotation of overlapping genes showed that DNA replication was the most significantly enriched pathway (ko03030) and biological process (GO: 0006260) (Table S4). GO enrichment analysis of all DEGs in three comparison groups showed that DEGs were mainly enriched in DNA replication initiation (GO: 0006270), DNA packaging complex (GO: 0044815), protein-DNA complex (GO: 0032993), MCM complex (GO: 0042555), mitotic cell cycle process (GO: 1,903,047), phenylpropanoid metabolic process (GO: 0009698), nucleosome (GO: 0000786), trihydroxystilbene synthase activity (GO: 0050350), microtubule binding (GO: 0008017) and tubulin binding (GO: 0015631) (Fig. S2a-c). KEGG pathway analysis divided DEGs into 135, 138, and 136 pathways, respectively (Table S5). Notably, plant hormone signal transduction was significantly enriched at three comparison groups, indicating that plant hormones played an important role in the regulation of peanut pod-growth. Moreover, MAPK signaling pathway-plant, starch and sucrose metabolism, and phenylpropanoid biosynthesis were also found to be enriched in three comparison groups (Fig. 3d).

### Key DEGs related to phytohormones biosynthesis and signaling pathways during the rapid -growth stage

Previous studies have shown that the biosynthesis process and signal-mediated transduction of auxin, CK and GA are related to cell division and cell expansion [32], thus affecting the size of plant fruit. The KEGG analysis presented in this study provides evidence for a significant change in the gene expression of auxin, CK and GA both in biosynthesis process and signal-mediated transduction pathway. Therefore, we focused on the DEGs involved in biosynthesis and signal transduction of these phytohormones.

**Fig. 4:**
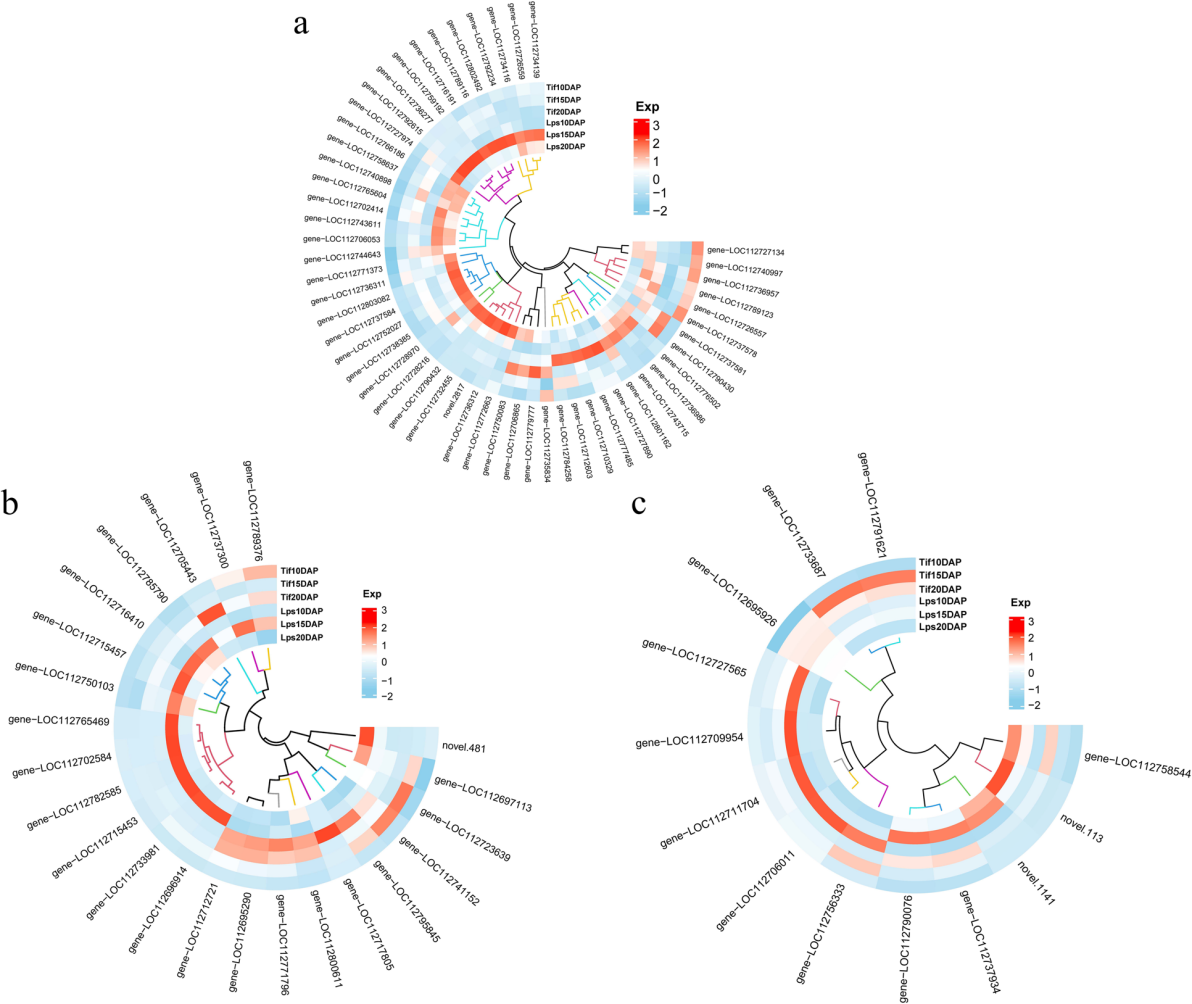
Expression profiles of the DEGs involved in biosynthesis and signaling transduction pathway of auxin, CK and GA. **a** Heatmap of DEGs related to the auxin biosynthesis and signaling pathway. **b** Heatmap of DEGs related to the CK biosynthesis and signaling pathway. **c** Heatmap of DEGs related to the GA biosynthesis and signaling pathway

Auxin is a well-known phytohormone that has a strong effect on cell enlargement and plant growth. There were 46 (25 up- and 21 down-regulated), 76 (35 up- and 41 down-regulated) and 63 (32 up- and 31 down-regulated) DEGs between Tif and Lps at 10DAP, 15DAP and 20DAP, respectively, participating in the auxin biosynthetic process, auxin -mediated signalling pathway and response to auxin (Table S6). Both TAA1 and TDC had positive regulatory effects on auxin biosynthesis. *Gene-LOC112801162* (TAA1) and *gene-LOC112727890* (TDC) were found to be up-regulated at 10DAP in Lps. The genes that participated in auxin-mediated signalling, namely, GH3 (auxin responsive GH3 gene family: *gene-LOC112737581*) and ARF (auxin response factor: *gene-LOC112743715*) were found to be differentially expressed at all stages. In Lps, the expression of *gene-LOC112737581* (GH3) was up-regulated at 10DAP and down-regulated at 15DAP and 20DAP, and *gene-LOC112743715* (ARF) was up-regulated at all stages. In addition, two ARFs (*gene-LOC112712603*, *gene-LOC112728970*) were found to be significantly up-regulated at 10DAP and *gene-LOC112803082* (ARF) was up-regulated at 15DAP (Fig. 4a).

There were 16 (9 up- and 7 down-regulated), 33 (14 up- and 19 down-regulated) and 30 (9 up- and 21 down-regulated) DEGs participating in the biosynthesis and signal transduction of CK, respectively (Table S6). Isopentenyltransferases (IPTs) are responsible for the bulk of CK biosynthesis [33]. *Gene-LOC112741152* and *gene-LOC112795845* were two DEGs encoding IPTs, both of which were significantly up-regulated in Lps at 10DAP. Another DEG: *gene-LOC112705443* (IPT) was significantly up-regulated at 15DAP. There are two types of response regulators (ARRs) in CK signaling pathway. Positive regulation of CK response by type B ARR protein. *Gene-LOC112771796* (B-ARR) and *gene-LOC112712721* (B-ARR) were up-regulated in Lps compared with Tif at 10DAP (Fig. 4b).

GA plays an important role in promoting cell division and elongation [34]. For the biosynthesis and signal transduction of GA, 43(25 up- and 18 down-regulated), 59(22 up- and 37 down-regulated) and 46(24 up- and 22 down-regulated) DEGs were involved in (Table S6). *ent*-kaurenoic acid oxidase (KAO), GA3-oxidase (GA3ox), GA 20-oxidase (GA20ox) and GA 2-oxidase (GA2ox) are key enzymes in GA biosynthesis. The results showed that *gene-LOC112756333* (KAO), *gene-LOC112695926* (KAO), *gene-LOC112706011* (GA20ox), *gene-LOC112711704* (GA20ox), *gene-LOC112709954* (GA3ox) and *gene-LOC112727565* (GA3ox) were all up-regulated in Lps at 10DAP (Fig. 4c).

### Quantitative analysis of phytohormones during early-growth stage of pods.

**Fig. 5:**
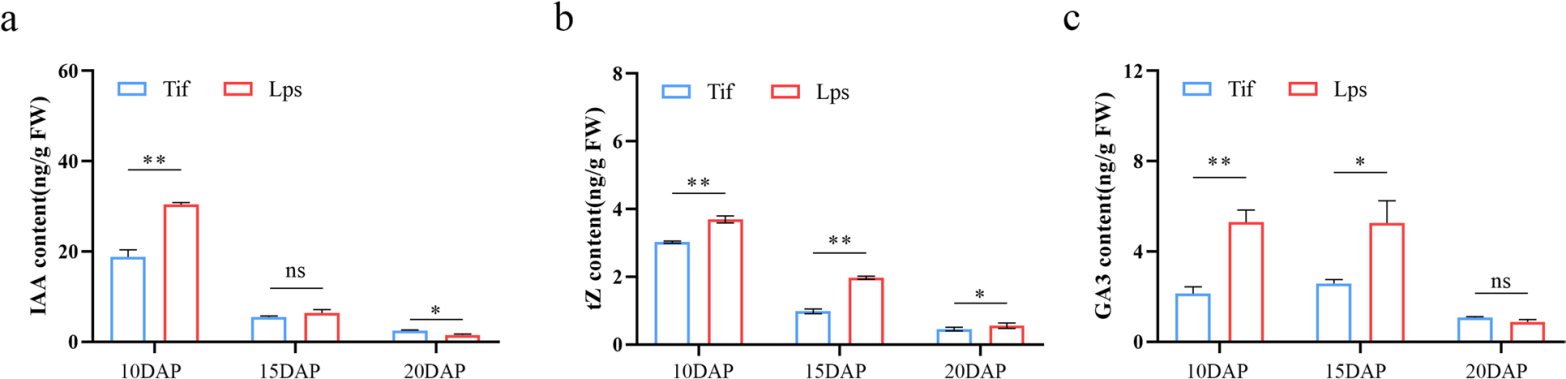
Auxin, CK and GA contents of Tif and Lps pods at 10DAP, 15DAP and 20DAP. **a** Indole-3-acetic acid (IAA) content. **b** trans-zeatin (tZ) content. **c** GA3 content. Error bar is SD. ** *p* < 0.01.* *p* < 0.05

The LC–MS/MS absolute quantification analysis of these phytohormones was performed since there were many DEGs in the biosynthesis of auxin, CK and GA. The contents of 26 auxins, 36 CKs and 12 GAs were detected during the early-growth stage (Table S7). Results suggested that the phytohormones contents of Tif and Lps were higher during the rapid-growth stage of peanut pods, and then decreased rapidly, which was consistent with the expression trend of related genes (Fig. 4, Table S8). We further compared the differences in the contents of IAA, tZ (*Trans* zeatin) and GA3 between Tif and Lps. At 10DAP, the content of IAA in Lps was significantly increased with a fold change (Lps/Tif) of 1.61 and a *P*-value of 0.0035. No significant difference in IAA contents between Lps and Tif at 15DAP (Fig. 5a). However, the content of IAA in Lps was significantly decreased with a fold change (Lps/Tif) of 0.61 and a *P*-value of 0.0017 at 20DAP. The tZ contents of Lps was significantly higher than that of Tif during the early -growth stage. The fold changes (Lps/Tif) of 10DAP, 15DAP and 20DAP were 1.22, 2.01 and 1.21, respectively (Fig. 5b). The contents of GA3 in Lps increased significantly during rapid-growth stage of pod with fold changes (Lps/Tif) of 2.48 and 2.04, respectively. At 20DAP, compared with Tif, the GA3 content of Lps was decreased, but no significant difference (Fig. 5c).

### Analysis of DEGs participating in cell division

According to the cytological observation results, we assumed that the larger pod size of Lps was associated with cell division. Hence, the DEGs related to cell division were investigated. The results showed that there were 57 (45 up- and 12 down-regulated), 146 (8 up- and 138 down-regulated) and 118 (9 up- and 109 down-regulated) DEGs at 10DAP, 15DAP and 20DAP, respectively (Table S9). Since cell division mainly occurs during the rapid-growth stage, we focus on the DEGs significantly up-regulated in Lps during this period. D-type cyclins are conserved in plants, known as sensors for growth conditions and trigger the G1/ S transition [35]. In the present study, *gene-LOC112747313* (log2FoldChange = 2.6617) encoding cyclin-D4 was significantly up-regulated at 10DAP. At 15DAP, *gene-LOC112754661* was most significantly up-regulated. Likewise, *gene-LOC112716260* (Cell division control protein) and three genes encoding dynamin-related protein (*gene-LOC112730699*, *gene-LOC112776526* and *gene-LOC112733381*) were found to be up-regulated significantly (Table 1).

**Table 1:** The top 5 significantly up-regulated DEGs in Lps at rapid-growth stage

Group	Gene ID	Log2FC	*p*-value	Description
Tif10DAP vs. Lps10DAP	*gene-LOC112747313*	2.66	1.50E-03	Cyclin-D4
	*gene-LOC112695821*	1.98	2.16E-07	Wee1-like protein kinase
	*gene-LOC112712603*	1.83	2.42E-04	Auxin response factor
	*gene-LOC112721292*	1.80	1.92E-08	Targeting protein for Xklp2
	*gene-LOC112766595*	1.67	2.54E-04	Cell division control protein 6
Tif15DAP vs. Lps15DAP	*gene-LOC112754661*	1.21	5.26E-12	Signal recognition particle receptor
	*gene-LOC112716260*	1.11	2.20E-07	Cell division control protein 48
	*gene-LOC112730699*	1.26	2.78E-11	Dynamin-related protein
	*gene-LOC112776526*	1.26	2.28E-09	Dynamin-related protein
	*gene-LOC112733381*	1.06	2.30E-06	Dynamin-related protein

### Validation of candidate DEGs by qRT-PCR analysis

**Fig. 6:**
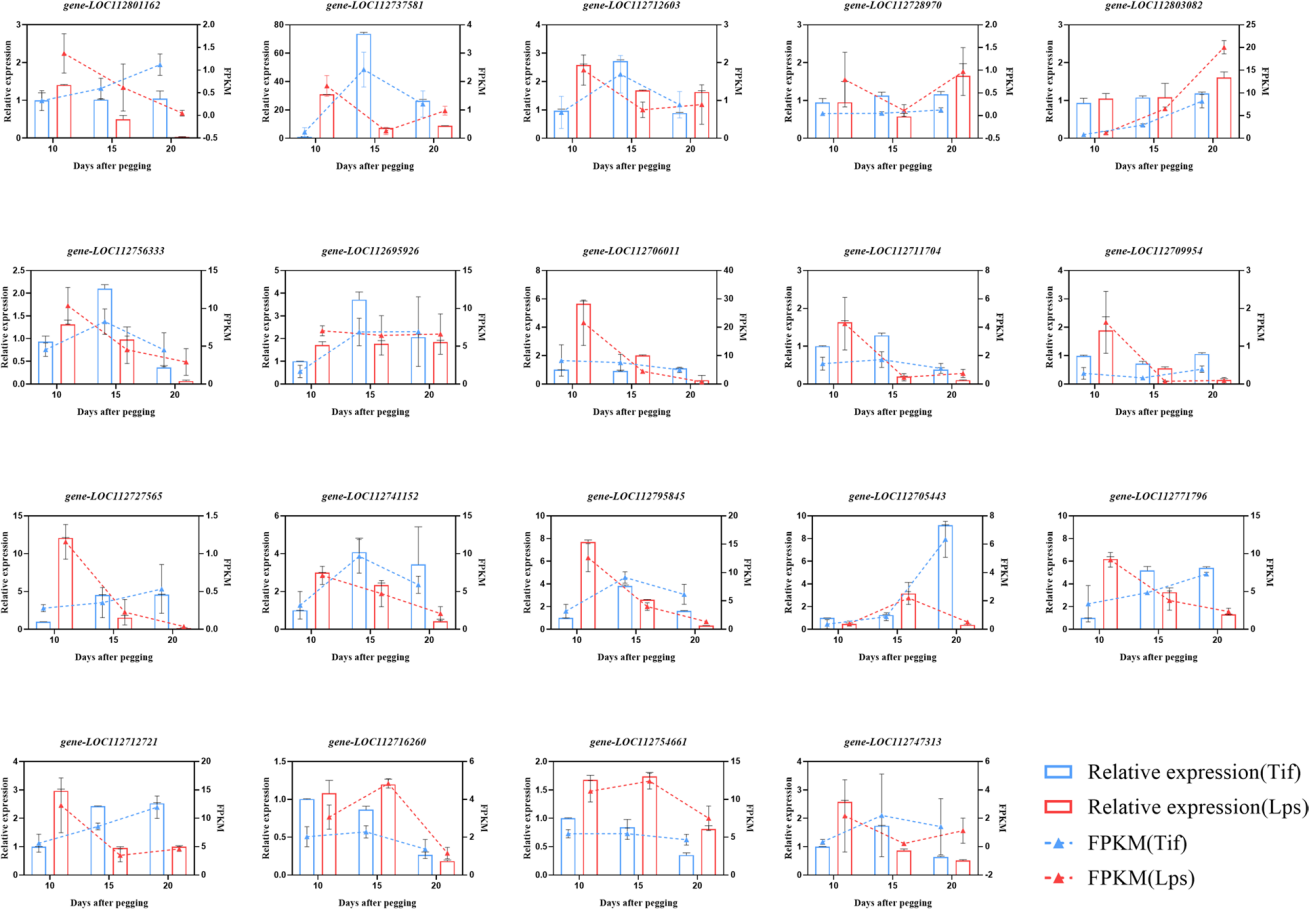
qRT-PCR verification of DEGs between Tif and Lps. The left y-axis shows the relative expression levels analyzed by qRT-PCR and the right y-axis shows the RPKM value analyzed by RNA-seq. Data represent the mean of three replicates ± SD

Five, five, and six genes involved in the biosynthesis and signal transduction of auxin, CK and GA and three genes related to cell division were selected, respectively, and qRT-PCR was used to analyze their expression to verify the transcriptome data sets from RNA-Seq. The qRT-PCR results for these 19 genes were in close agreement with the corresponding relative transcript abundances obtained from RNA-Seq (Fig. 6), validating the reliability of RNA-seq results.

## Discussion

The development of peanut pods affects the final yield. In this process, the peanut shell develops first and acts as a protective and perceived organ to ensure the normal development of seeds [36]. Larger pods (shells) are the basis for obtaining larger seeds. However, the development of pods and seeds is not synchronized, and large pods do not necessarily obtain large seeds. For example, Ca^2+^ deficiency can cause empty pods at the seed-filling stage [37]. Thus, the relationship between pod size and shelling percentage should be synthetically considered in breeding efforts. Peanut forms pegs after fertilization and the pod-development process is triggered only if elongating pegs penetrate into the soil [38]. In order to explore the developmental pattern of peanut pods, we analyzed the length and the width of Tif and Lps pods during five stages after pegging. According to the logistic growth-function, the pod expansion could be divided into three stages. The first stage was the gradual-growth stage of the pod. During this period, the sizes of pods increased slowly. Subsequently, the pod size increased rapidly and the pod entered the rapid-growth stage (10DAP-15DAP). The next is the slow-growth stage, when the pod size increased slowly and reached its final size. Previous studies showed that the final size of plant fruit is determined during early growth stage [39, 40]. In this study, our findings suggested that the final size of peanut pod may be determined during rapid-growth stage.

The fruit size is mostly determined by cell number rather than cell size [41, 42]. The anatomical structure of Tif and Lps pods was compared first in our study. In agreement with previous studies, our results suggested that the cell-number was the critical factor causing the difference in pod sizes between Tif and Lps. However, some previous studies on the peanut pod size showed that the cell-area was the main factor affecting pod size [3, 29]. On one hand, this could be due to the fact that the cellular basis of pods differs amongst peanut varieties. On the other hand, the development-pattern of pod cells during rapid-growth stage (especially 15DAP) was not investigated in the studies mentioned above. During the fruit development process in plant, the cell division occurs first. Once the cell division is completed, the cell expansion begins [43]. The cell-number of Tif and Lps pods increased rapidly from 10 to 15DAP and slowed down after 15DAP. In the aspect of cell-area, there was no significant increase until 15DAP, but increased significantly thereafter 15DAP. All these results suggested that the cell-number increase was the main developmental events during the rapid-growth stage of peanut pods. After the rapid-growth stage the cell number no longer increased, but the cell area began expanding. Therefore, the peanut pod-size was determined by the cell number during the rapid-growth stage.

To probe the molecular mechanisms underlying the determination of peanut pod sizes, we performed transcriptional profiling of peanut pods during early-growth stage. The result showed that the number of DEGs between Tif and Lps was increased significantly during the rapid-growth stage. KEGG pathway enrichment analysis revealed that many of these DEGs were enriched in starch and sucrose metabolism and plant hormone signal transduction, etc. Starch and sucrose metabolism are energy sources for plant-growth [44, 45]. SUS catalyzes reversible reaction to decompose sucrose into UDPG [46], thus providing substrate for cellulose biosynthesis [47]. Moreover, GAUT is an enzyme promoting pectin synthesis [48, 49]. In this study, most of *SUSs* and *GAUTs* in Lps exhibited higher gene expression levels at 10DAP (Fig. 6). This could lead to accumulate more of cellulose and pectin during the rapid-growth stage in Lps. Plant cell division requires coordinated synthesis and deposition of new walls between two daughter cells [50]. It is well known that cellulose and pectin are the major components of plant cell walls. Hence, the up-regulation of these genes was related to more cells in Lps during rapid-growth stage.

**Fig. 7:**
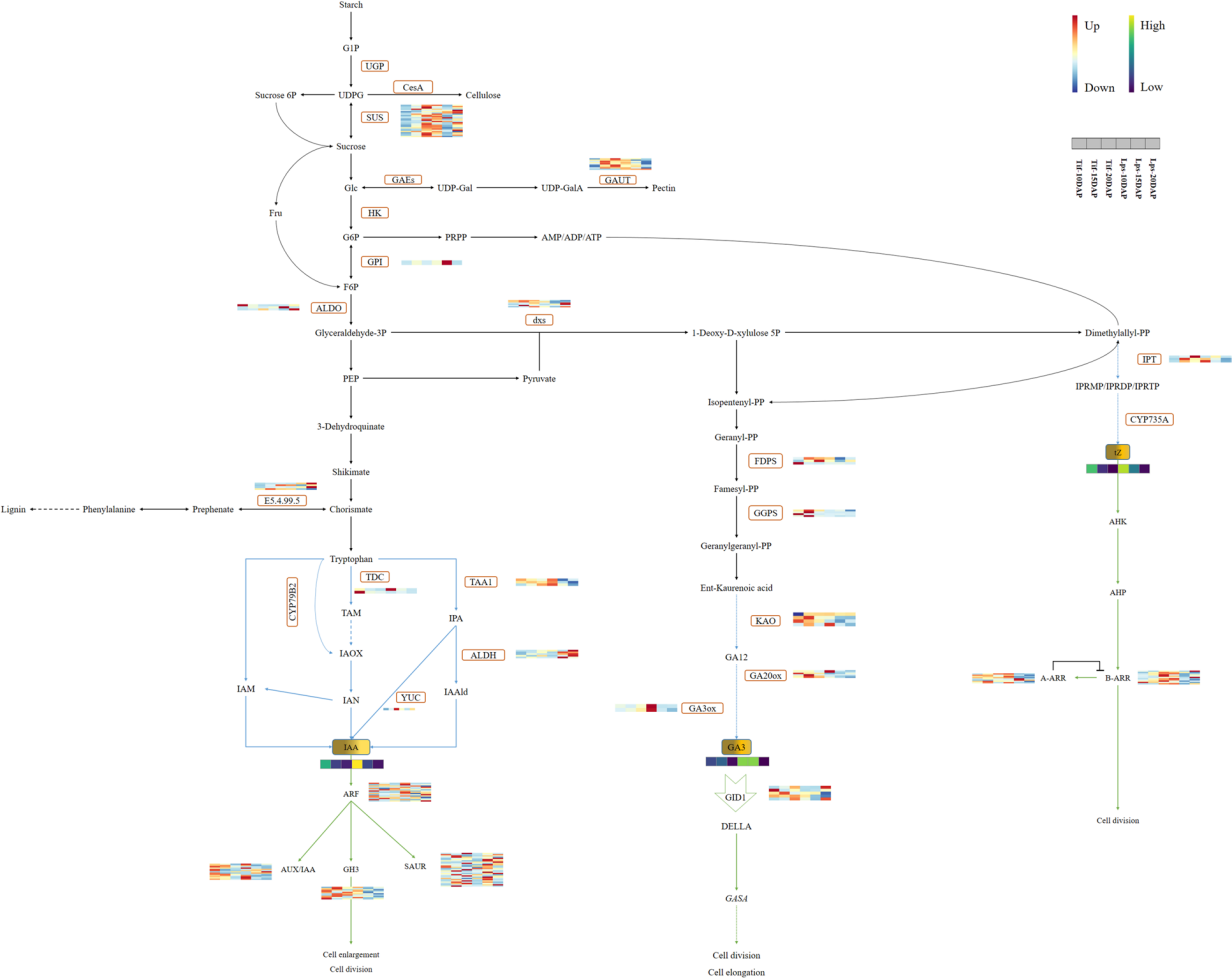
Phytohormones biosynthesis and signal transduction pathways. Biosynthesis and signal transduction are represented by blue and green lines, respectively. The solid arrow indicates a direct step, and the broken arrow indicates an indirect step. FPKM values of the genes were Z-score standardized. For genes, the key is located at right side with FPKM values increasing from skyblue to red. For phytohormones, the color scale indicates low (darkblue) to high (yellow) content

Phytohormones, including auxin, CK and GA, play an important role during the early-growth stage of peanut pods [28]. These phytohormones affect pod size mainly by regulating cell division and expansion. In this study, we found that the cell-number in the rapid-growth stage was the main factor determining peanut pod sizes. Meanwhile, this critical period was also the stage where the phytohormones content of pods was the highest. Therefore, we speculated that the difference in phytohormones content during this period was the main reason for the difference in the cell-number of Tif and Lps. Tryptophan is an important precursor for auxin biosynthesis [51]. Tryptophan is first converted by the TAA family of amino transferases to indole-3-pyruvic acid (IPA), and then IAA is produced from the IPA by the YUC family of flavin monooxygenases [52–54]. In Arabidopsis, the research has shown that auxin levels can be regulated by modulation of *TAA1* gene transcription [55]. In this study, the expression pattern of *gene-LOC112801162* (TAA1) was consistent with the changing trend of IAA content, indicating that the differentially expressed of *TAA1* resulted in the different content of IAA between Tif and Lps. *ARF* and *GH3* play downstream roles in IAA signaling pathway and are responsible for plant growth [34]. Previous studies suggested that auxin regulates seed size mainly through auxin response factors (ARFs) [56]. Meanwhile, *GH3* is regulated by *ARF* [57] and participates in tissue or organ development in leguminous plants [58]. Luo et al. identified 63 *AhARF* genes from an allotetraploid peanut cultivar, of which *AhARF14/26/45* were significantly associated with root development [59]. In Lps, two ARFs (*gene-LOC112712603* and *gene-LOC112728970*) were significantly up-regulated at 10DAP, and *gene-LOC112743715* (ARF) was up-regulated at all stages. Furthermore, *gene-LOC112737581* (GH3) was up-regulated at 10DAP (Fig. 7). These DEGs were predicted to be important genes involved in peanut pod-growth.

The ability of CK to promote cell division was first discovered more than sixty years ago [60]. *Trans*-zeatin (tZ) is the main active form of CK in most plants [61]. Isopentenyl transferases (IPTs) are involved in the first step in CK biosynthesis by catalyzing isopentenyl transfer from dimethylallyl diphosphate to adenine nucleotides [62]. In peanut, overexpression of the *IPT* gene improves drought tolerance and increases yield [63]. In this study, two *IPTs* (*gene-LOC112741152*, *gene-LOC112795845*) were significantly up-regulated in Lps at 10DAP, which led to more accumulation of CK in Lps. There are two types of type-A *Arabidopsis* response regulators (ARRs) involved in CK signaling: type-A ARRs and type-B ARRs [64]. The type-B ARR proteins are activated by changes in their phosphorylation state, which positively regulates CK response by activating transcription of their downstream targets [65]. At present, the genome-wide identification of type-B ARR family members has not been reported in peanut. In this study, *gene-LOC112771796* (B-ARR) and *gene-LOC112712721* (B-ARR) were significantly up-regulated in Lps at 10DAP (Fig. 7).The up-regulation of these DEGs associated with CK was speculated to be responsible for the difference in the cell-number between Tif and Lps.

GA is involved in various aspects of plant growth and development. *ent*-kaurenoic acid oxidase (KAO), GA 20-oxidase (GA20ox) and GA 3-oxidase (GA3ox) are key enzymes in GA biosynthesis [66]. It has been reported that the gene *NA* encode KAO in pea, and the *na* mutant showed a GA-deficient dwarf phenotype [67]. In Arabidopsis, *GA20ox* regulates plant growth and development by modulating GA levels [68]. Overexpression of *GA20ox* can enhance seed size [69]. *PsGA3ox1* transgenic plants were reported to have longer pea fruits [70]. In this study, *gene-LOC112756333* (KAO), *gene-LOC112706011* (GA20ox), *gene-LOC112711704* (GA20ox), *gene-LOC112709954* (GA3ox) and *gene-LOC112727565* (GA3ox) were significantly up-regulated in Lps at 10DAP (Fig. 7). In a recent study, Wang et al. [29] reported that *GA20ox* genes were significantly down-regulated in a peanut mutant with a small pod, which was consistent with our results. This suggests that further study of *GA20ox* genes in peanut is necessary. Overall, these key DEGs may positively regulate pod size through modulation of GA biosynthesis.

**Fig. 8:**
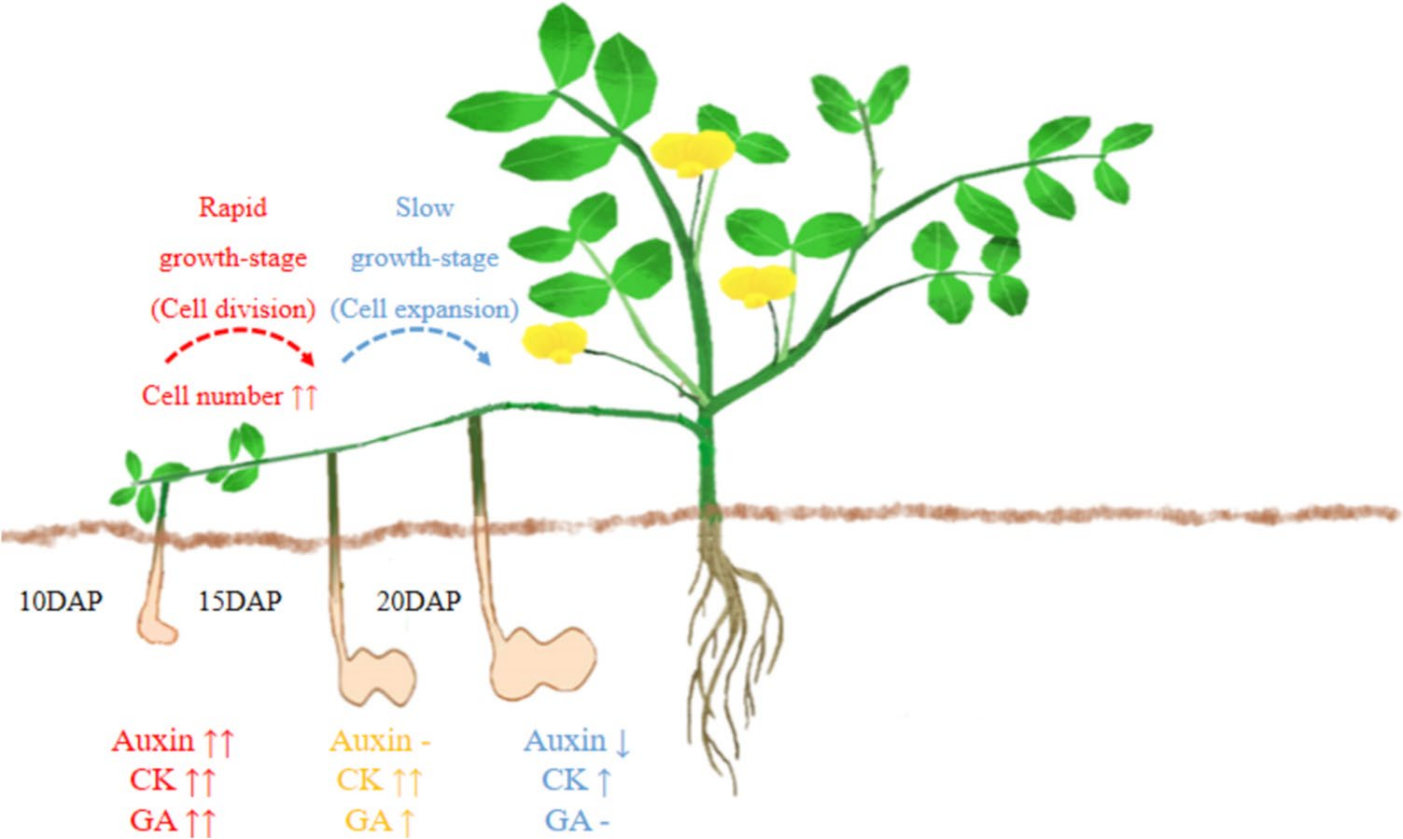
Schematic representation of early-growth stage. Cell division was mainly carried out from 10 to 15 DAP, and cell expansion was mainly carried out from 15 to 20DAP. The changes of auxin, CK and GA content in Lps compared to Tif showed in the figure.↑↑and ↑indicate the significant increase (*p* < 0.01) and (*p* < 0.05), respectively.—indicates no significant difference between Tif and Lps

In the present study, the auxin, CK, and GA contents measured by LC–MS/MS were consistent with the RNA-Seq analysis results. The contents of these phytohormones were higher during the rapid -growth stage and decreased significantly after 15DAP, indicating that the regulation of phytohormones on peanut pod-growth was mainly during the rapid-growth stage. In addition, we found that only the tZ contents were extremely significant different between Tif and Lps at both 10DAP and 15DAP, which suggested that CK might be a decisive factor contributing to the difference in peanut pod sizes. In summary, we proposed a simple model for peanut pod-growth during early stage regulated by phytohormones (Fig. 8). In this model, the difference in phytohormones levels is due to DEGs associated with phytohormones biosynthesis. Subsequently, changes in phytohormones levels and phytohormones signal transduction related DEGs lead to differences in cell division of peanut pod. Finally, the cell number during the rapid-growth stage determines the pod size. However, if we want to construct a comprehensive development-network in peanut pods, we may also need more studies such as the changes in proteomic and metabolomic, and genome-wide identification of key gene families (such as type-B ARR), which is one of our future works.

## Methods

### Plant materials

Two peanut varieties with contrasting pod size were used in this study. The larger pod size line Lps is the backbone parent used for breeding high yield peanut germplasm in Northern China, showing exciting potential for breeding purposes in the long-term breeding work. The smaller pod size variety is Tifrunner (the reference genome of peanut, hereafter referred as Tif.). Both Tif and Lps were planted in the same areas (Laixi, Shandong, China). The peg that had not penetrated the soil of Tif and Lps were tied with colored tags (cotton thread), respectively. Peg penetrated into the soil on different days were marked with different colored tags. After marking, the soil was covered to ensure that the pegs were buried. Subterranean pods were collected from plants grown in the field at 10, 15, 20, 25 and 30DAP.

### Trait measurements and calculations

Pod length and width were measured by Vernier caliper (five biological replicates for each material). The development pattern of pod length and width fitted by Logistic growth model [71, 72] with CurveExpert 1.4 software, the formula is: ${\varvec{Y}}={\varvec{K}}/(1+{\varvec{a}}{{\varvec{e}}}^{-{\varvec{b}}{\varvec{t}}})$** (1)**. In the formula (1): Y is the length or width at any time (cm • pod^−1^); K is the maximum length or width (cm • pod^−1^); t is the number of days after pegging; a and b are undetermined coefficients.

The velocity function of Logistic growth process can be obtained by calculating the first derivative of formula (1):${\varvec{V}}\boldsymbol{ }\left({\varvec{t}}\right)={\varvec{d}}{\varvec{y}}/{\varvec{d}}{\varvec{t}}={\varvec{K}}{\varvec{a}}{\varvec{b}}{{\varvec{e}}}^{-{\varvec{b}}{\varvec{t}}}/{(1+{\varvec{a}}{{\varvec{e}}}^{-{\varvec{b}}{\varvec{t}}})}^{2}$** (2)**. In the formula (2): V (t) is the rate of development; the following formulas are obtained by the first-order derivation and the second-order derivation of formula (2) and making it equal to 0: ${{\varvec{t}}}_{{\varvec{m}}{\varvec{a}}{\varvec{x}}}={\varvec{l}}{\varvec{n}}{\varvec{a}}/{\varvec{b}}$; ${{\varvec{t}}}_{1}={\varvec{l}}{\varvec{n}}{\varvec{a}}-1.317/{\varvec{b}}$; ${{\varvec{t}}}_{2}={\varvec{l}}{\varvec{n}}{\varvec{a}}+1.317/{\varvec{b}}$. In these formulas: t_max_ is the occurrence time of maximum growth rate; t_1_ and t_2_ are the start and end time of rapid-growth stage. *t*_*1*_ and *t*_*2*_ divided the process of peanut pods expansion into gradual-growth stage, rapid-growth stage and slow-growth stage of length (or width).

### Cytological observation and analysis

Peanut pods were collected at 10DAP, 15DAP and 20DAP, and immediately fixed in formalin-aceto-alcohol (FAA), dehydrated with a graded series of ethanol (75%, 85%, 90%, 95%, and 2 × 100%), infiltrated with 100% xylene and embedded in paraffin. Serial 6-μm sections were cut with an RM2016 microtome (Leica, Shanghai, China), stained with toluidine blue, and visualized with a Nikon ECLIPSE E100 microscope (Nikon Instruments, Japan). Image J software was explored to measure cell number and size of the parenchymal cell of exocarp.

### RNA-Seq

We selected 10 representative pods from Tif and Lps for each biological library construction (three biological replicates for each time point). RNA-Seq was performed using RNA extracted from peanut pods using RNAprep Pure Plant Plus Kit (TIANGEN BIOTECH, Beijing, China). To meet the requirements of RNA library construction, the RNA concentration and RNA integrity were detected by RNA Nano 6000 Assay Kit of the Bioanalyzer 2100 system (Agilent Technologies, CA, USA) and Qubit® RNA Assay Kit in Qubit®2.0 Flurometer (Life Technologies, CA, USA), respectively. Library quality was assessed on the Agilent Bioanalyzer 2100 system (Agilent Technologies, Palo Alto, California, USA). The cDNA libraries were sequenced on the Illumina sequencing platform by Metware Biotechnology Co., Ltd. (Wuhan, China). Use fastp (version 0.19.3) to filter the original data, mainly to remove reads with adapters clean reads were mapped to the reference genome sequence (*Arachis hypogaea* cv. Tifrunner) (https://www.peanutbase.org/data/public/Arachis_hypogaea/). All subsequent analyses are based on clean reads. Use feature Counts v1.6.2 to calculate the gene alignment, and then calculate the FPKM of each gene based on the gene length. DESeq2 v1.22.1 was used to analyze the differential expression between the two groups, and the *P* value was corrected using the Benjamini & Hochberg method. The corrected *P* value and fold change |log2| are used as the threshold for significant differential expression. To identify differentially expressed genes (DEGs), a stringent value of |fold change|> 2 and corrected *p*-value < 0.05 were used as thresholds. The enrichment analysis is performed based on the hypergeometric test. For KEGG, the hypergeometric distribution test is performed with the unit of pathway; For GO, it is performed based on the GO term.

### Quantitative analysis of phytohormones by LC–MS/MS

Peanut pods collected at 10DAP, 15DAP and 20DAP were used to detect the content of phytohormones. Phytohormones including auxin, CK and GA were detected by MetWare (http://www.metware.cn/) based on the AB Sciex QTRAP 6500 LC–MS/MS platform. Each treatment contained three replicates. Determination by the method described previously [73].

### Quantitative real-time PCR (qRT-PCR)

The samples used for qRT-PCR analysis were the same as RNA-seq. Gene-specific primers for qRT-PCR were shown in Table S1. The qRT-PCR was conducted using a SYBR Premix Ex Taq™ kit (TaKaRa, Dalian, China) following the manufacturer’s instructions. The amplification conditions were as follows: predenaturation at 95 °C for 10 min, denaturation at 95 °C for 15 s, and annealing and extension at 60 °C for 30 s. Fluorescence signals were collected during annealing and extension and the whole process was repeated for 40 cycles. To determine the relative expression of each gene among different samples, the 2^−△△Ct^ method was used along with the internal reference actin gene to normalize the results.

### Statistical analysis

Data was analyzed using Microsoft Excel and plotted using GraphPad Prism 8.0.2 and OriginPro 2021b software. Statistical analyses were performed using SPSS 17.0 software (SPSS, Inc.). Cell statistics using CaseViewer 2.4 and ImageJ software. Circle diagrams and bubble plots were prepared using OmicShare tools (www.omicshare.com/tools). Heatmaps were created by R software using “ComplexHeatmap” and “circlize” package.

## Supplementary Information

**Additional file 1: Fig. s1.**

**Additional file 2: Fig. s2.**

**Additional file 3: Table S1.** Gene-specific primers for qRT-PCR.

**Additional file 4: Table S2.** Logistic growth function.

**Additional file 5: Table S3.** GO enrichment analysis.

**Additional file 6: Table S4.** Enrichment analysis of overlapping DEGs.

**Additional file 7: Table S5.** KEGG enrichment analysis.

**Additional file 8: Table S6.** DEGs related to phytohormones.

**Additional file 9: Table S7.** Quantitative analysis of phytohormones.

**Additional file 10: Table S8.** DEGs consistent with the change of phytohormones.

**Additional file 11: Table S9.** DEGs participating in cell division.

## Data Availability

The RNA-Seq data have been submitted to the NCBI Sequence Read Archive (SRA; http://www.ncbi.nlm.nih.gov/sra/) database with the accession number PRJNA828366.
