# Supplementary figures and images for: The determination of peanut (Arachis hypogaea L.) pod-sizes during the rapid-growth stage by phytohormones
Source: BMC Plant Biol. 2023 Jul 26;23:371. doi: 10.1186/s12870-023-04382-w (PMC10369843; doi:10.1186/s12870-023-04382-w)

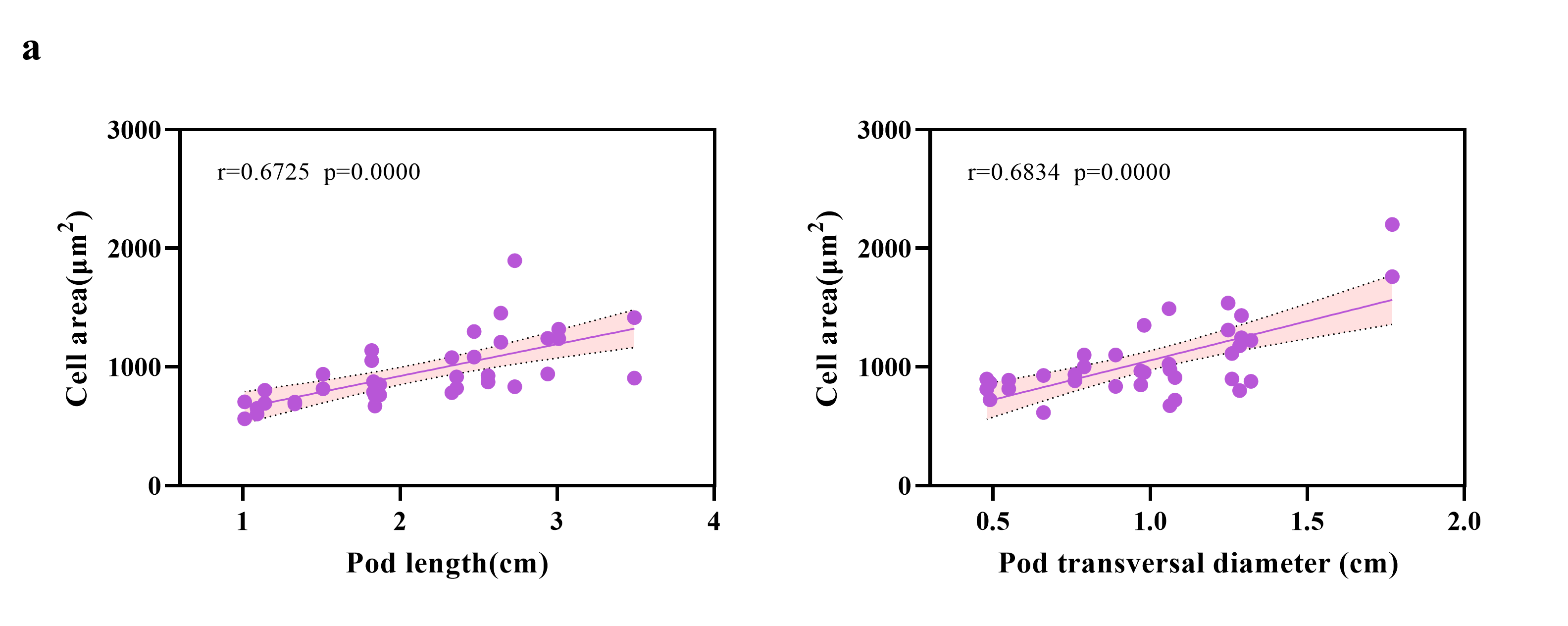

Supplement: Supplementary file 1 — Additional file 1: Fig. s1. [file 12870_2023_4382_MOESM1_ESM.tif]

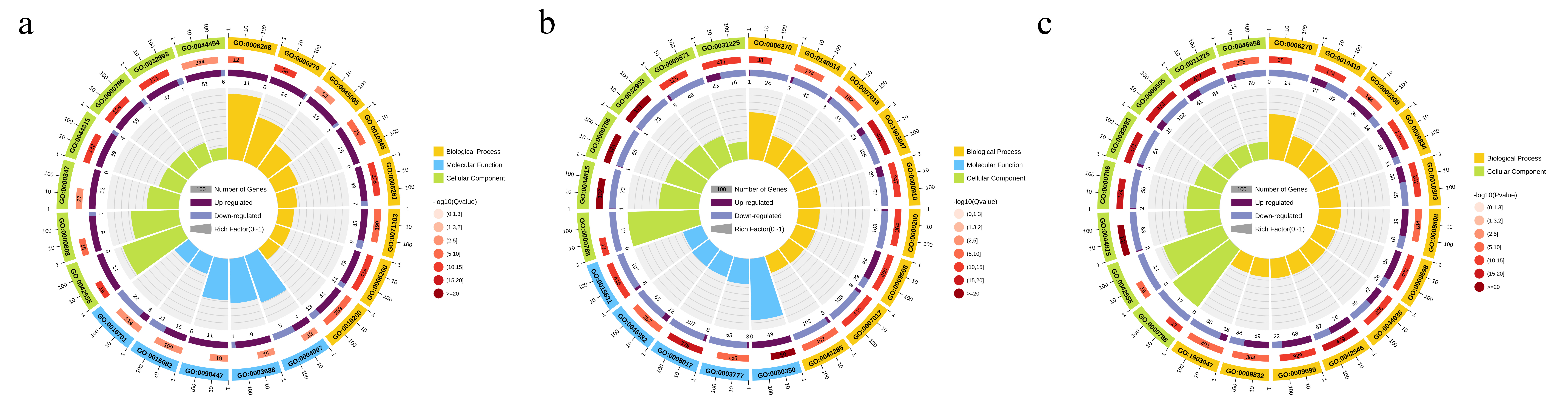

Supplement: Supplementary file 2 — Additional file 2: Fig. s2. [file 12870_2023_4382_MOESM2_ESM.jpg]
